# Relationship between Nutritional Status, Food Consumption and Sarcopenia in Post-Stroke Rehabilitation: Preliminary Data

**DOI:** 10.3390/nu14224825

**Published:** 2022-11-15

**Authors:** Mariacristina Siotto, Marco Germanotta, Alessandro Guerrini, Simona Pascali, Valeria Cipollini, Laura Cortellini, Elisabetta Ruco, Yeganeh Manon Khazrai, Laura De Gara, Irene Aprile

**Affiliations:** 1 IRCCS Fondazione Don Carlo Gnocchi ONLUS, 50143 Florence, Italy; 2 Department of Science and Technology for Humans and the Environment, Università Campus Bio-Medico di Roma, 00128 Rome, Italy; 3 Unit of Food Science and Nutrition, Department of Science and Technology for Humans and the Environment, Università Campus Bio-Medico di Roma, 00128 Rome, Italy

**Keywords:** post-stroke, sarcopenia, bioelectrical impedance analysis, rehabilitation, functional recovery, malnutrition: nutrition, Geriatric Nutritional Risk Index, plate waste, food consumption, nutritional intake

## Abstract

After a stroke, patients can suffer from sarcopenia, which can affect recovery. This could be closely related to an impairment in nutritional status. In this preliminary analysis of a longitudinal prospective study, we screened 110 subjects admitted to our rehabilitation center after a stroke. We then enrolled 61 patients, who underwent a 6-week course of rehabilitation treatment. We identified a group of 18 sarcopenic patients (SG), according to the European Working Group on Sarcopenia in Older People 2 (EWGSOP2), by evaluating muscle strength with the handgrip test, and muscle mass with bioelectrical impedance analysis (BIA). With respect to the non-sarcopenic group (NSG), the SG at admission (T0) had worse muscle quality, according to the BIA-derived phase angle, and a lower score of MNA^®^-SF. In contrast to the NSG, the SG also exhibited lower values for both BMI and the Geriatric Nutritional Risk Index (GNRI) at T0 and T1. Moreover, 33% of the SG had a major risk of nutrition-related complications (GNRI at T0 < 92) and discarded on average more food during the six weeks of rehabilitation (about one-third of the average daily plate waste). Of note is the fact that the Barthel Index’s change from baseline indicated that the SG had a worse functional recovery than the NGS. These results suggest that an accurate diagnosis of sarcopenia, along with a proper evaluation of the nutritional status on admission to rehabilitation centers, appears strictly necessary to design individual, targeted physical and nutritional intervention for post-stroke patients, to improve their ability outcomes.

## 1. Introduction

Stroke is the leading cause of adult disability [1,2] and the second largest cause of death worldwide, with a high burden on patients, their families, and healthcare systems [3].

After a stroke, patients can be affected by sarcopenia, which is a progressive and generalized skeletal muscle disorder [4]. Sarcopenia is defined as either “primary“, when correlated with a physiological manifestation of the ageing process of the body, or “secondary”, when associated with activity, disease or nutrition, although there is not a clear clinical demarcation [5]. Disease-related sarcopenia accelerates the progression of muscle atrophy, and becomes part of the disease process itself. In this context, “stroke-related” sarcopenia has been proposed as a result of gradual and progressive muscular atrophy brought on by neurological disorders, inflammation, and immobility [6,7]. Recent studies have shown that stroke survivors with sarcopenia have a bad recovery [8,9,10,11,12], whereas an improvement in sarcopenia corresponds to a better functional outcome [13].

Nutritional impairment is a common risk for stroke survivors, and malnutrition has been identified as a frequent complication, correlated with poor daily-living function and quality-of-life levels [14,15,16]. Several stroke-related conditions contribute to malnutrition, such as dysphagia, restricted upper limb movement, visuospatial impairment, increased catabolic processes, gastrointestinal dysfunction and depression [17,18,19].

Moreover, sarcopenia and an inadequate dietary status have a close relationship: malnutrition or a limited nutritional intake of calories and macronutrients lead to muscle decline, and may contribute to or aggravate post-stroke sarcopenia [6]. Therefore, early nutritional status assessment and, if necessary, nutritional intervention are required to improve sarcopenic-stroke outcomes.

In recent years, there has been a rapid increase of studies regarding the role of nutrition in rehabilitation outcomes after a stroke [20,21,22]. However, only few studies examined nutritional status correlated to sarcopenia or body-mass composition in post-stroke subjects [17,23,24,25].

Sarcopenia and malnutrition are also frequently undiagnosed and untreated during or after rehabilitation. High-quality clinical evidence regarding nutrition in sarcopenic post-stroke patients during rehabilitation is urgently required. Additionally, it would be crucial to broaden the investigation into patients’ nutritional behavior, including screening surveys, anthropometric measurements, parameters based on biological measurements, and the recording of food waste and dietary intake of patients.

The aim of this study’s preliminary analysis is to assess in a cohort of post-stroke patients during a 6-week course of rehabilitation treatment: (i) the presence of sarcopenia according to EWGSP2 guidelines; (ii) the nutritional status measured by different parameters and the food waste in the sarcopenic and non-sarcopenic individuals; (iii) the comparison of the rehabilitation outcomes between sarcopenic and non-sarcopenic subjects.

## 2. Materials and Methods

### 2.1. Study Design and Participants

This longitudinal, prospective study is still ongoing (NUTRISTROKE study, clinical trials identifier: NCT04923165). The preliminary data presented herein, are based on a cohort of 61 patients with a first stroke, admitted to our rehabilitation department between June 2021 and May 2022. They were consecutively enrolled and analyzed at admission (T0) and after a 6-week course of rehabilitation treatment (T1).

The inclusion criteria were as follows: (i) first ischemic or hemorrhagic stroke, documented using magnetic resonance imaging (MRI) or computed tomography (CT); (ii) age between 18 and 85 years; (iii) time since the stroke onset less than 6 months; (iv) sufficient cognitive and language skills to understand the instructions for the administration of the assessment scales, and to sign the informed consent.

The exclusion criteria were as follows: (i) a previous stroke; (ii) behavioral and cognitive disorders and/or reduced compliance, interfering with active therapy or with understanding and signing informed consent; (iii) presence of pacemakers (for interference with bioimpedance measures).

The study design was approved by the Ethical Committee of Don Carlo Gnocchi Foundation, Milan, Italy on 14 October 2020 (FDG_6_14/10/20). Written informed consent was obtained from all patients after a detailed explanation of the study’s aims and rehabilitation protocols.

### 2.2. Rehabilitation Treatment

Patients underwent a rehabilitation program including conventional physical therapy, which was performed 6 days a week for 45 min. It comprised sensorimotor stimulation, passive, active-assisted and active mobilizations, exercises for muscle-strength recovery, stretching, functional and task-oriented training), proprioceptive exercises, postural passages and transfers, sitting and standing training, motor-coordination and balance training, walking training and activities of daily-living recovery training. Moreover, all patients performed a robotic treatment of the upper limb, 5 times a week, each session lasting 45 min, using a set of robotic devices. The robotic treatment was based on the use of 4 robotic devices: Motore (Humanware Srl, Pisa, Italy), and Amadeo, Diego and Pablo (Tyromotion GmBH, Graz, Austria) [26,27]. During the upper-limb robotic treatment, patients performed both motor and cognitive tasks, and the devices provided visual and auditory feedback, to help them.

### 2.3. Clinical Assessment and Activity of Daily-Living Assessment

Demographic, anamnestic, and clinical data were recorded on admission (T0). Disease burden was measured with the 56-point Cumulative Illness Rating Scale (CIRS) [28].

Functional independence was evaluated by the modified Barthel Index (BI), an ordinal scale used to measure performance in activity of daily living (ADL) [29]. Patients were evaluated at T0 and re-evaluated after 6 weeks of rehabilitation treatment (T1), to assess the effect of the provided treatment. The presence of dysphagia was recorded at T0, without describing the severity. Patients admitted were not characterized by clinical conditions that prevented them from feeding themselves. In addition, no patients required enteral nutrition, (e.g., nasogastric tube, gastrostomy, etc.), or parenteral nutrition.

### 2.4. Malnutritional Risk Screening

Patients were screened at T0 for risk of malnutrition, according to GLIM criteria [30], using the Mini Nutritional Assessment Short-Form (MNA^®^-SF), a simple, quick and easy nutritional tool based on the full Mini Nutritional Assessment (full MNA^®^). The MNA^®^-SF includes six geriatric-specific assessment questions related to nutritional and health conditions. Patients were considered to be of “normal nutritional status” if the MNA^®^-SF screening score value was from 12 to 14, at “risk of malnutrition” with a value between 8 and 12, and “malnourished” with a score of less than 7.

### 2.5. Nutritional Status Assessment

The assessment of the nutritional status during the study was performed by means of: (i) anthropometric measurements (ii) the Geriatric Nutritional Risk (GNRI), and (iii) a daily estimation of food consumption

(i). Anthropometric measurements comprised body height and weight evaluations. Height was recorded at T0 in all patients able to stand, reporting data in meters (m), up to the nearest 0.1 cm. In other cases, the referred height was recorded and checked with the knee-height [31]. Body weight was checked at T0 and T1, as well as weekly, for monitoring any consistent weight changes. Weight was evaluated to the nearest 0.1 kg on a calibrated weighing scale or, for non-autonomous patients, on a weighing chair. Measurements were taken after overnight fasting in the morning, without heavy clothing or shoes. The Body Mass Index (BMI) at T0 and at T1 was then calculated and expressed in kg/m^2^.
(ii). We calculated the GNRI at admission (GNRI T0) and after 6 weeks of rehabilitation (GNRI T1), to assess the risk of nutritional-related complication. Values of GNRI < 92 are considered indices of impaired nutritional status [32]. The GNRI was calculated following the formula [33]:

GNRI = [1.489 × albumin (g/L)] + 41.7 × (weight/WLo)]

Specifically, serum albumin levels were measured using a bromocresol colorimetric assay (Diacron, GR, Italy), and tested on an integrated analytical photometer (Free Carpe Diem, Diacron, GR, Italy). To standardize the assessment of those biochemical variables that are affected by the circadian cycle and food intake, blood samples of patients were collected in the early morning (7:30–9:00 a.m.) after overnight fasting. Sera samples were separated by centrifugation (3000 rpm, 10 min, and 4 °C), and then divided into 0.5 mL aliquots and rapidly stored at −80 °C. Subjects’ samples and reference samples were thawed just before the assay. All the analyses of serum albumin were performed in duplicate, both at T0 and at T1.

WLo corresponds to ideal body weight, according to the Lorentz formula that considers the patient’s height [cm] and sex. WLo for men: (height − 100) − ((height − 150)/4); WLo for women: (height − 100) − ((height − 150)/2).

(iii). We estimated the amount of food intake during the 6 weeks of study, through the visual estimation of plate waste [34,35,36]. The meals were formulated and prepared by the company canteen, according to the Italian guidelines “National Recommended Energy and Nutrient Intake Levels for the Italian population” (LARN, [37]). For dysphagic patients, the meals were served as texture-modified food, or fluid. The nurses and speech therapists reported the meal waste (breakfast, lunch, and dinner), for 6 days a week, for 6 weeks (108 meals in total for each patient), assigning a score from 0 to 4 on a 5-point scale (0 = none wasted; 1= ¼, 2 = ½, 3 =¾, and 4 = all wasted). The daily average plate-waste score was then calculated for all the meals consumed (n = 108) for all patients.

### 2.6. Sarcopenia Assessment

Subjects were divided into the Sarcopenic Group (SG) and the Non-Sarcopenic Group (NSG), on the basis of the European Working Group on Sarcopenia in Older People revised guideline (EWGSOP2) [38].

The identification of sarcopenia was conducted through a two-step approach, where each step was performed when the previous one was positive:

- Muscle-strength observation.
- Evaluation of muscle quantity.

Muscle strength of non-hemiparetic hand was assessed by the handgrip test using a hand-held digital dynamometer (Citec, C.I.T Techincs, Haren, The Netherlands), that measures the maximum isometric strength of the hand and the forearm muscles. Patients were measured in a sitting position with elbows flexed at 90°, shoulders adducted, and forearms in a neutral position, without support. Patients were instructed to squeeze the dynamometer as hard as possible three times, and the mean value was reported in kilograms [39]. in line with the EWGSOP2 criteria, we identified patients with probable sarcopenia as those with a handgrip strength of less than 27 kg in men and 16 kg in women.

Muscle-mass quantity and quality was estimated by bioelectrical impedance analysis (BIA), carried out with a tetrapolar whole-body BIA device (BIA 101 anniversary sport edition, Akern, Florence, Italy). Electrodes were positioned on the healthy hemisoma, and all patients were examined in supine position with their four limbs evenly apart. Muscle quantity was measured by appendicular skeletal muscle mass (ASMM), estimated from proprietary manufacturer algorithms using Bodygram Plus software (Akern, Florence, Italy). In line with the EWGSOP2 criteria, the ASMM was divided by the height squared (ASMM/h^2^; kg/m^2^). The EWGSOP2 sarcopenia cut-off points for low muscle-mass quantity were <7 kg/m^2^ for men and <5.5 kg/m^2^ for women [38]. As an index of muscle quality, we measured the phase angle which is derived from resistance (R_z_) and reactance (X_c_) obtained from the BIA analysis and expressed in degrees. The normal value suggested by the manufacturer is between 5° and 9°, and its variability may depend on age, gender, ethnicity, body composition, level of physical activity, and adiposity [40].

### 2.7. Statistical Analysis

Descriptive statistics were used to express demographic and clinical characteristics of patients, with numerical data expressed as the mean (SD), and categorical data presented as counts and percentages.

To compare the nutritional status variables in SG and NSG, we used the Mann–Whitney U test, or the chi-squared test, as appropriate.

To investigate if the sarcopenic status could influence the recovery of ability in the ADL, we first computed the change from the baseline of the BI (ΔBI = BI_T1_ − BI_T0_); then, we performed an analysis of covariance (ANCOVA) to determine the effect of sarcopenia on recovery, as measured by the ΔBI. The baseline value of the BI, as well as clinical and demographic variables (age, sex, time since stroke, type of stroke, and hemiparesis side) were considered as covariates.

For all the statistical analysis, a p value lower than 0.05 was deemed significant. Statistical analysis was performed using IBM SPSS Statistics for Windows (Version 28.0. IBM Corp, Armonk, NY, USA).

## 3. Results

### 3.1. Participants and Baseline Characteristics

For the study, 110 patients were screened. According to the inclusion criteria, 61 patients (30 men and 31 women; mean age 68 ± 11 years) were enrolled and evaluated at baseline (T0) and after a six-week course of rehabilitation treatment (T1). Only three patients did not complete the follow-up, because of adverse clinical conditions.

Table 1 reports the baseline characteristics (demographic and clinical features, disability assessment, anthropometric measurements, and nutritional status) of the enrolled sample.

Patients were found to be at high risk of malnutrition, as assessed by the MNA^®^-SF questionnaire (Table 1). In particular, 32 subjects were malnourished (with a score lower than 7), 26 were at risk of malnutrition (with a score between 8 and 11), and only 3 were not at risk of malnutrition (with a score between 12 and 14).

### 3.2. Sarcopenia Analysis

**Figure 1:**
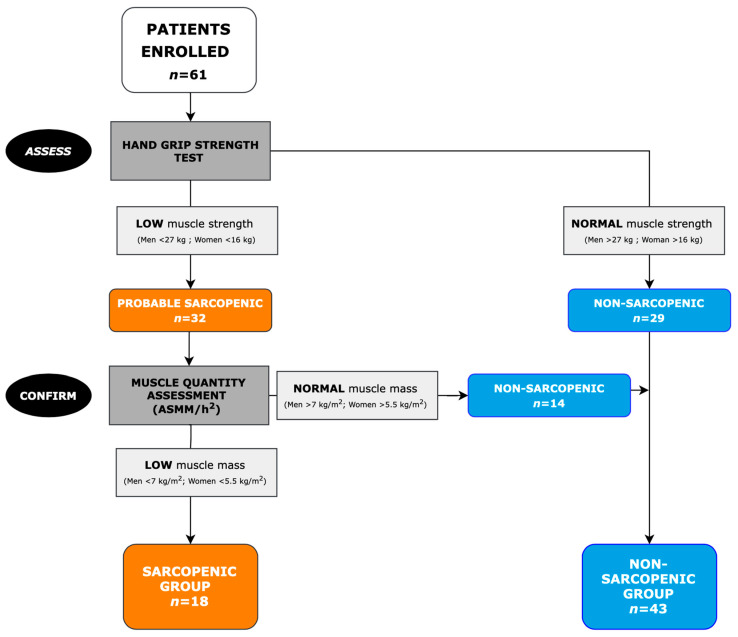
EWGSOP2 algorithm for case finding and diagnosis of sarcopenia at baseline in the sample group. Patients enrolled (*n* = 61) were assessed by handgrip test and the diagnosis in patients with probable sarcopenia (*n* = 32) was confirmed by muscle-quantity assessment using bioelectrical impedance analysis (BIA), identifying a Sarcopenic Group (SG) of 18 subjects and a Non-Sarcopenic Group (NSG) of 43 subjects.

In our sample, the EWGSOP2 algorithm identified 32 probable sarcopenic subjects, 18 of whom were confirmed as sarcopenic (Figure 1).

The phase-angle values measured with bioelectrical impedance analysis, were lower in the SG, with respect to the NSG (4.8° ± 1.5°; 5.8° ± 2.5°; *p* = 0.025).

### 3.3. Nutritional Status

Table 2 reports the differences in nutritional-status variables in the SG and NGS. Patients in the SG showed a statistically significant lower MNA^®^-SF score at T0. Moreover, the values of BMI and GNRI at both the timepoints (T0 and T1) were lower in sarcopenic subjects. Additionally, at the baseline, 33% of SG and only 10% of NSG were at risk of impaired nutritional status (GNRI < 92) (*p* = 0.030). Food wasted by the SG was higher than that wasted by the NSG during the rehabilitation period (Table 2). In fact, the SG had an average daily-discharge score of 1.10, which corresponds to approximately one-third of the plate not consumed; for the NSG the score was 0.72, corresponding to one-fifth of the plate not consumed.

### 3.4. Functional Recovery

**Figure 2:**
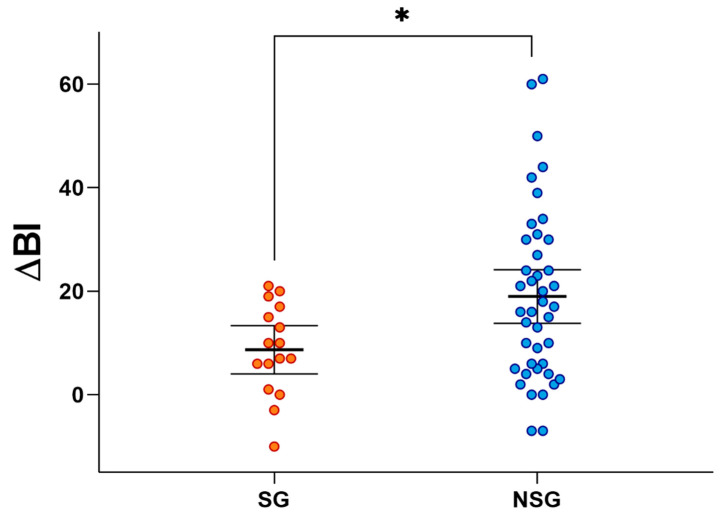
Distribution of the change from baseline of the modified Barthel Index (ΔBI) after 6 weeks of rehabilitation in Non-Sarcopenic Group (NSG, *n* = 42) and Sarcopenic Group (SG, *n* = 16). Mean bars and 95% CI are reported. * Refers to the statistically significant difference (*p* value = 0.038).

After adjustment for age, sex, time since stroke, type of stroke, hemiparesis side and BI at T0, there was a statistically significant difference in the ΔBI mean values of the two groups, with a lower value in the SG than in the NSG (8.7 ± 8.8 vs. 18.9 ±16.6; *p* = 0.038; Figure 2).

## 4. Discussion

The main finding of these preliminary data is that post-stroke subjects with confirmed sarcopenia showed a worse muscle quality and a worse nutritional status. In fact, the SG had lower values of phase angle, of MNA^®^-SF at admission, and lower values of BMI and GNRI, both at T0 and at T1; 33% of the SG had a major risk of nutritional-related complication at admission. Additionally, plate waste registered in during the six weeks of hospitalization revealed that sarcopenic patients discarded, on average, more food. Moreover, the Barthel Index’s change from baseline indicated that the SG had a worse functional recovery than the NGS.

According to the EWGSOP2 guidelines, the post-stroke patients enrolled in this study were clinically characterized by the typical symptoms and signs that could lead to the suspicion of sarcopenia. In line with a recent meta-analysis [41], we confirmed the diagnosis of sarcopenia in 30% of the subjects, through BIA. By passing a small current through the body and determining the impedance as a result, the BIA can be used to indirectly determine body composition [33]. The gold-standard methods for estimating muscle mass or muscle quantity are: magnetic resonance imaging (MRI), computed tomography (CT), and dual-energy X-ray absorptiometry (DXA); however, these methods are very costly, are not portable, and require highly qualified personnel to run the equipment [38]. Despite a lower accuracy with respect to gold-standard methods, BIA analysis is widely used for muscle-mass or muscle-quantity measurements. Because it is minimally invasive and an easy-to-use tool, even during daily clinical practice [42].

In our study, the muscle quality of sarcopenic patients was worse, as evidenced by lower phase-angle values (4.8° ± 1.5° in SG vs. to 5.8° ± 2.5° in the NSG), which is in line with the data reported in a recent review on sarcopenia in geriatric subjects [40]. Muscle quality does not have a universally agreed definition, but its evaluation describes the complex intramuscular changes at tissue and cellular level associated with muscle performance [43]. For this reason, recent evidence suggests that the assessment of muscle quality could be more useful in the assessment of the effect of rehabilitation treatment [44,45]. Irisawa et al. reported that 179 post-stroke patients, after four weeks of rehabilitation, showed an increase in phase angle (4.2° at T0 and 4.5° at T1), which was positively correlated with an improvement of ADL measured with the Functional Independence Measure (FIM) [21], while muscle mass did not change and did not correlate with functional recovery. In a different study, 499 people undergoing post-stroke rehabilitation showed a positive correlation between phase angle and the return of physical function, as measured by FIM-motor scores at discharge and FIM-motor score gain [46]. Based on this evidence, additional studies in post-stroke patients during rehabilitation are necessary, to confirm whether there is a correlation between muscle quality and recovery, and whether phase angle can predict clinical outcome.

We found a correlation between sarcopenia and an altered nutritional status. The MNA^®^-SF at T0 score indicated that almost all participants in this study were at risk of malnutrition; this score in the SG was even lower (Table 2). However, although MNA^®^-SF is validated and is included in GLIM criteria for the assessment of those at risk of malnutrition, other reliable indices have been employed, such as the GNRI, which is a biological index of nutritional status, calculated with systemic albumin measurements and ideal body weight, and is a simple and accurate nutritional tool [33]. GNRI has a prognostic value in describing the nutritional status and nutritional-related complications in hospitalized elderly patients [47]. In fact, after adjusting for age, sex, and cancer, a GNRI value below 92 was significantly associated with worse MNA^®^ scores, and lower values for the following: weight, BMI, mid-arm circumference, calf circumference, and serum levels of total protein, albumin, and prealbumin. It was also an independent predictor of three- and six-month mortality [47]. Moreover, Kang et al. demonstrated that in patients with acute ischemic stroke, GNRI was associated with a poor prognosis [48].

In this study, the mean GNRI at T0 and T1 in the SG was lower than in the NSG (Table 2). At admission, a higher number of sarcopenic patients had GNRI values under 92 (33% in the SG vs. 9.3% in the NSG; Table 2), which refers to major nutrition-related risks, and therefore is very likely to incur an adverse outcome.

Another important result was that the SG had lower BMI both at T0 and at T1, and wasted more food than the NSG (one-third of average-daily-plate wasted by the SG, with respect to one-fifth wasted by the NSG). Although dysphagia could partly explain this result, there were no differences in the proportion of dysphagic people in the two groups. Consequently, we decided to analyze the food waste of each patient, to understand whether they were completing their meals. The results from these preliminary data strongly support the need for a future study specifically designed to evaluate the intake of macro- and micronutrients of post-stroke patients. In fact, analyzing a correct dietary intake in post-stroke patients while they are rehabilitating, could clarify whether a sarcopenic patient actually had a reduced nutritional intake. Moreover, it would be important to establish if the sarcopenia condition itself contributed to patients eating less. Additionally, food waste is strictly related to the problem of malnutrition among hospitalized patients, but it also has financial and environmental impacts that should not be underestimated [49].

It is noteworthy that the SG showed a poorer functional recovery as measured by ΔBI, in addition to a worse nutritional status (Figure 2). This agrees with recent studies which have focused on the role of sarcopenia and body composition during recovery in post-stroke patients admitted to rehabilitation units. Post-stroke survivors with sarcopenia showed higher levels of impairment, as measured by FIM at discharge [8], and by the modified Barthel Index [9]. Another study showed that the presence of decreased muscle mass in terms of skeletal muscle index (SMI) measured by BIA, and a lower handgrip strength, were correlated with negative effects on functional recovery in subacute stroke patients [10,12]. Furthermore, Matsushita et al. demonstrated that patients with improvement from sarcopenia showed higher FIM at discharge [13].

These studies suggest that more effort should be made to ameliorate the sarcopenia in post-stroke patients, and to allow them a better functional recovery by combining targeted nutritional intervention with physical rehabilitation treatment, in order to contrast sarcopenia in post-stroke patients hospitalized for a long time [17,23].

Nutritional intake in sarcopenia post-stroke subacute patients has not been well investigated, and there are limited nutritional data available throughout rehabilitation. One study found that providing stored energy contributes to weight gain and increases skeletal muscle-mass, and that it takes approximately 9600 kcal of energy to improve 1 kg of body weight in underweight patients [50]. Another study pointed out that protein supplementation may enhance neurological recovery in subacute patients with ischemic stroke [51]. In the research, it was shown that physical exercise and a healthy diet greatly reduced the detrimental effects of comorbidity on stroke patients’ impairment [52]. A very recent clinical randomized-control-trial demonstrated that supplementation of whey protein and Vitamin D in post-stroke patients ameliorated muscle quality [24]. More studies are necessary to assess the correct approach to treating malnourished sarcopenic post-stroke patients in rehabilitation.

The major limit of this preliminary study regards the registration of food waste which did not consider the nutritional intake in terms of calories, proteins, carbohydrates and lipids, and specific micronutrients. For this reason, we could not state whether the patients had an adequate intake to achieve daily nutritional requirements. Another limitation is that it was not possible to discuss whether patients’ malnutrition was related to comorbidities, due to the limited number of patients. A future study is planned, to monitor the exact intake of nutrients in all patients admitted for rehabilitation after a stroke. Moreover, in cases of impaired nutritional status, it would be suitable to examine the association with the presence of comorbidities. The fact that patients were only enrolled in this research’s single rehabilitation facility is another drawback. However, we recently launched a multicentered study, to deal with this restriction and to increase the sample size.

## 5. Conclusions

According to these preliminary data, nutritional condition and functional recovery were worse in sarcopenic post-stroke patients admitted to a rehabilitation center, compared with non-sarcopenic ones. The diagnosis of sarcopenia, together with a proper assessment of nutritional status at admission, is strictly necessary in order to design individual targeted physical and nutritional intervention in post-stroke patients, to improve their clinical outcomes.

## Figures and Tables

**Table 1:** Baseline characteristics of the sample (*n* = 61).

Baseline Characteristics	Mean (±SD) or Number (%)
Age (years)	68 ± 11
Sex	
Men	30 (49.2%)
Women	31 (50.8%)
Index stroke type	
Ischemic	42 (70%)
Hemorrhagic	18 (30%)
Affected side	
Right	30 (49.2%)
Left	31 (50.8%)
Days from stroke onset to enrollment	105 ± 61
Neglect	11 (18%)
Aphasia	12 (19.6%)
Smokers and former smokers	25 (41%)
Dysphagia	24 (39.3%)
Comorbidities	
Hypertension	54 (88.5%)
Type 2 Diabetes	21 (34.4%)
Dyslipidemia	31 (50.8%)
Hearth disease (heart failure or prior heart attack)	7 (11.5%)
Atrial fibrillation	7 (11.5%)
Prior Cancers	6 (9.8%)
Peripheral Vascular Disease (PVD)	12 (19.7%)
Chronic Obstructive Pulmonary Disease (COPD)	7 (11.5%)
Thyroid Disease	14 (23%)
More than 2 comorbidities	47 (77%)
Cumulative Illness Rating Scale (CIRS) CIRS severity CIRS comorbidity	2.4 ± 0.4 6.0 ± 1.9
Activities of Daily Living (ADL) Assessment	
Modified Barthel Index (0–100)	45 ± 18
Anthropometric Measurements	
Height (m)	1.66 ± 0.11
Weight (kg)	68.0 ± 17
BMI (kg/m^2^)	24.4 ± 5.3
Nutritional status	
Mini Nutritional Assessment Short-Form (MNA^®^-SF)	7 ± 2
Albumin (g/L)	37.4 ± 6.5
Geriatric Nutritional Risk Index (GNRI)	104.9 ± 14.1

**Table 2:** Nutritional status variables in Sarcopenic Group (SG) and Non-Sarcopenic group (NSG). Data are reported as mean ± standard deviation or number percentage (%). *P* values refer to the Mann–Whitney U test or the χ^2^ test, as appropriate.

	Sarcopenic Group (SG)	Non-Sarcopenic Group (NSG)	*p* Value
MNA^®^-SF (T0)	6 ± 2	8 ± 2	<0.001 **
BMI (T0)	20.6 ± 2.2	26.0 ± 5.5	<0.001 **
BMI (T1)	20.7 ± 2.3	26.0 ± 5.2	<0.001 **
GNRI (T0)	96.9 ± 15.2	108.2 ± 12.4	0.010 *
GNRI (T1)	100.1 ± 11.2	109.7 ± 12.3	0.015 *
Delta GNRI	2.2 ± 9.0	1.1 ± 6.9	0.309
GNRI < 92 (T0)	33%	9.3%	0.030 *
GNRI < 92 (T1)	29%	9.7%	0.073
Dysphagia	50%	35%	0.207
Plate waste (daily average)	1.10 ± 0.64	0.72 ± 0.57	0.020 *

* *p*-value < 0.05; ** *p*-value < 0.005.

## Data Availability

The data supporting the findings of this study are available from the corresponding author upon reasonable request.
